# Réalités pour une stratégie de lutte contre la drépanocytose dans la région africaine de l’Organisation Mondiale de la Santé: Compte rendu du 7^e^ Symposium International du Réseau d’Étude de la Drépanocytose en Afrique Centrale (REDAC https://www.redacnetwork.org/) Antananarivo, Madagascar. 13 - 15 juin 2018

**DOI:** 10.48327/PSMF-0S04

**Published:** 2021-02-18

**Authors:** B. Ngasia, L. Tshilolo, G. Loko, C. Vodouhe, G. Wamba, J.-P. Gonzalez

**Affiliations:** Les auteurs sont membres du Réseau d’Etude de la Drépanocytose en Afrique Centrale (REDAC).; 1 Centre Hospitalier Alliance, et Programme National de Lutte Contre la Drépanocytose (PNLCD), Kinshasa, République Démocratique du Congo; 2 Centre de Formation et d’Appui sanitaire (CEFA) et Centre Hospitalier Monkole, République Démocratique du Congo; 3 Centre Hospitalier de la Martinique, Guadeloupe, France; 4 DORYS, Association de Lutte Contre la Drépanocytose, Strasbourg, France; 5 Service Médical, Centre Hospitalier d’Essos, Caisse Nationale de Prévoyance Sociale, Yaoundé, Cameroun; 6 Center of Excellence for Emerging & Zoonotic Animal Disease, CEEZAD, Kansas State University, Office Park, 1800 Kimball Ave, Suite 130, Manhattan, KS, 66502, USA

**Keywords:** Drépanocytose, REDAC, Afrique, Madagascar, Sickle cell disease, REDAC, Africa, Madagascar

## Abstract

Le Réseau d’Étude de la Drépanocytose en Afrique Centrale ou REDAC, est un réseau de chercheurs africains, européens et américains qui a comme objectif la lutte contre la drépanocytose. Ses congrès se déroulent chaque année dans les pays partenaires avec des symposium internationaux qui alternent avec des ateliers de travail. Le REDAC permet aux pays hôtes d’obtenir des autorités locales une réelle implication dans la lutte à travers des résolutions en phase avec les stratégies nationales de lutte. Le Septième Symposium International du REDAC s’est tenu en 2018 à Antananarivo, Madagascar sous l’égide du Ministre Malgache de la Santé, et des Autorités Malgaches du Sénat. Le thème choisi était celui des stratégies de lutte contre la drépanocytose recommandées par l’OMS. Les communications ont porté sur le dépistage néonatal, le diagnostic précoce, la prise en charge du drépanocytaire et les nouvelles thérapeutique (greffe de moelle, thérapie génique, et traitement par l’hydroxyurée).

## Abstract (en)

The Network for the Study of Sickle Cell Disease in Central Africa or REDAC, is a network of African, European and American researchers whose aim is to combat sickle cell disease. Its congresses take place every year in the partner countries with international symposia alternating with workshops. REDAC enables host countries to obtain from local authorities a real involvement in the fight through resolutions in line with national strategies of fight. The Seventh International Symposium of REDAC was held in 2018 in Antananarivo, Madagascar under the auspices of the Malagasy Minister of Health and the Malagasy Senate Authorities. The theme chosen was that of strategies to combat sickle cell disease recommended by the WHO. The presentations focused on neonatal screening, early diagnosis, management of sickle cell disease and new therapies (marrow transplant, gene therapy and treatment with hydroxyurea).

## Introduction

Le Septième Symposium International du Réseau d’Étude de la Drépanocytose en Afrique Centrale (REDAC) s’est tenu du 13 au 15 juin 2018 à Antananarivo, Madagascar. Les symposiums du REDAC se déroulent dans un des pays d’accueil partenaires et alternent de façon bisannuelle avec un atelier de travail. En 2017, le Deuxième Atelier du REDAC, s’était déroulé à Kinshasa, République Démocratique du Congo, sur les thèmes du diagnostic et du suivi des drépanocytaires en Afrique centrale [1]. Le REDAC est un réseau africain de chercheurs regroupant les chercheurs africains, européens et américains avec comme objectif commun la lutte contre la drépanocytose. Les congrès du REDAC en plus de l’opportunité de partage d’expérience entre scientifiques, malades et familles des malades, permettent aux pays hôtes d’obtenir des Autorités locales, une réelle implication dans la lutte à travers les résolutions produites et en phase avec les plans stratégiques nationaux.

**Fig. 1:**
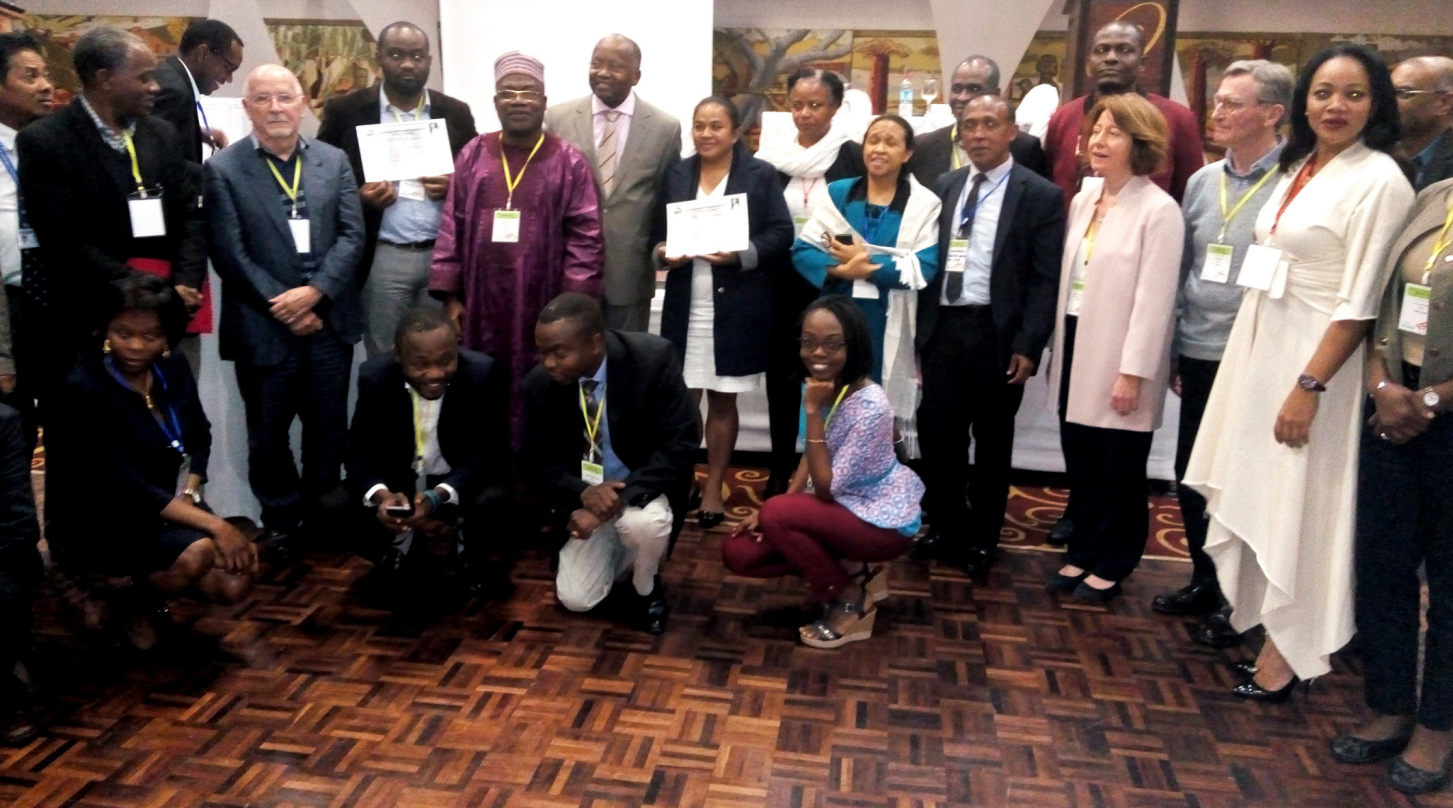
Participants du 7^e^ symposium du REDAC Participants of the 7^th^ REDAC Symposium

Ce 7^e^ symposium, placé sous le haut patronage du Président de la République malgache et le Ministère de la Santé a accueilli 218 participants. Plusieurs experts venus d’Afrique, d’Europe, d’Inde, du Moyen Orient ainsi qu’une forte participation malgache s’étaient réunis sur le thème principal qui était celui de partager les expériences de chacun et proposer des pistes des solutions adaptées à l’Afrique, où vivent plus de la moitié des personnes atteintes de la drépanocytose. Ces journées ont été très enrichissantes avec une cérémonie d’ouverture marquée par la présence du Ministre malgache de la Santé, du Représentant de l’OMS à Madagascar, l’ambassadeur de France à Madagascar, une délégation de la Principauté de Monaco, les Autorités malgaches du sénat. Au total plus de 70 communications ont été présentées en 12 sessions portant sur différents thèmes: du dépistage néonatal et diagnostic précoce à la prise en charge incluant les nouvelles stratégies thérapeutiques actuelles; à savoir l’accès à la greffe de la moelle et la thérapie génique en Afrique. Le premier jour a connu les interventions portant sur les outils de diagnostic et de dépistage néonatal, le suivi et la prévention des complications. Le dépistage néonatal et précoce précédé par des sensibilisations, constitue la clé de la meilleure prise en charge de cette maladie.

Les exposés du deuxième jour ont porté sur l’expression clinique de la drépanocytose, les indications et complications liées à la transfusion alors que les interventions du troisième jour avaient porté sur la lutte contre les infections et le paludisme, l’anesthésie et chirurgie, la greffe et thérapie génique, la recherche et la formation, la drépanocytose et les sociétés et; pour conclure, avec une table ronde sur ces journées et l’octroi du prix Dominique Labie à deux jeunes médecins (une malgache et un congolais de la RDC) engagés dans la lutte contre la drépanocytose. Lors de ce congrès, la stratégie de lutte contre la drépanocytose préconisée par l’OMS Afrique lors des assises de Malabo a été analysée en détail, laquelle admet cette maladie comme étant un problème majeur de santé publique, et par conséquent, recommande aux nations africaines le dépistage néonatal comme une des priorités dans la prise en charge de cette maladie. On estime à 380 000 le nombre de nouveau-nés qui naissent chaque année avec la forme homozygote, 80 % vivent en Afrique [3].

## La confÉrence inaugurale

La conférence inaugurale a porté sur la stratégie de la réduction du fardeau de la drépanocytose en Afrique. Cette stratégie qui passe par une prise de conscience du fardeau de la maladie, la formation des prestataires, le dépistage néonatal et une prise en charge précoce à travers une pénicilline-prophylaxie, une vaccination spécifique, le recours à l’hydroxyurée, le dépistage et la prise en charge des complications, et la surveillance; la mise en place des normes de soins et la formation du personnel; la transfusion sanguine, le partenariat de recherche et le plaidoyer. S’agissant de l’épidémiologie actuelle de la drépanocytose, on note un changement d’une distribution du gène Beta S avec une augmentation du nombre des porteurs qui est observée en Europe, ceci à cause des mouvements migratoires des populations.

## Mini symposium terumo BCT

La déléguée a présenté la place et l’impact de la pratique des échanges érythrocytaires automatisés dans le traitement du patient drépanocytaire. Terumo BCT est un leader international dans les domaines du traitement des composants sanguins, de l’aphérèse thérapeutique et des technologies cellulaires et résolument engagé dans le domaine de l’aphérèse thérapeutique et de la collecte cellulaire.

## L’institut europÉen pour la coopÉrationetledÉveloppement

L’IECD, dans le cadre de son programme multi-pays sur l’amélioration de la prise en charge de la drépanocytose au Cameroun, au Congo Brazzaville, en RDC et à Madagascar, a présenté les résultats du dépistage néonatal réalisé dans les quatre pays. Ce dépistage qui a permis de dépister près de 100 000 nouveau-nés, et d’avoir ainsi des données sur les enfants homozygotes régulièrement suivi à travers les centres pilotes. De plus, les parents bénéficiaient ainsi de l’éducation thérapeutique et d’accompagnement.

## Bilan oms drÉpanocytose

Malgré l’existence de quelques programmes de dépistage néonatal ciblé en Afrique, le bilan de l’OMS drépanocytose en Afrique subsaharienne reste mitigé (OMS, 2010). En effet, la drépanocytose demeure à ce jour encore, une maladie oubliée et ignorée non seulement par les populations, mais en particulier par les décideurs, plus focalisés sur les autres maladies non transmissibles (cardiovasculaires; cancers). Les propositions ou recommandations formulées par l’OMS à l’endroit des pays africains, ne sont pas appliquées, il n’existe pas un dépistage néonatal systématique, couplé aux vaccins et à la pénicilline.

## Comment rÉduire les fardeaux de la drÉpanocytose en afrique?

Dans sa conférence inaugurale, le Président du REDAC, a présenté les différents défis de l’Afrique face à la drépanocytose: le manque des programmes de dépistage néonatal de la drépanocytose, la limitation de l’accès à une bonne prise en charge en milieux ruraux, le manque de formation du personnel soignant sur la maladie, la non-disponibilité de l’hydroxyurée et le manque de moyens. Ce sont les grands défis qui restent à relever si on veut réduire le fardeau de cette maladie, responsable de la mortalité élevée parmi les enfants de moins de 5 ans. La réduction de ce grand fardeau passe par les principaux axes suivants: la connaissance et la sensibilisation sur cette maladie; la formation du personnel soignant sur la prise en charge; le diagnostic correct de la maladie; le dépistage néonatal (DNN) et la surveillance; le renforcement des centres de prise en charge; l’accès au traitement, à une transfusion sécurisée et à l’hydroxyurée; la recherche scientifique, les partenariats et le plaidoyer.

L’expérience française a montré que nous assistions à une transition épidémiologique à cause de l’amélioration de la nutrition et du meilleur contrôle des maladies infectieuses, diminuant ainsi le taux de mortalité infantile. Dans ce contexte, la relative prévalence des maladies génétiques a tendance à augmenter en particulier dans les pays ayant un fort taux de population porteuse du gène beta S.

## Outils de diagnostic et dÉpistage nÉonatal suivi et prÉvention des complications

Le représentant du Cameroun a présenté les résultats de la 1^re^ phase 2015-2017 du projet pilote du dépistage néonatal de la drépanocytose au Cameroun et le suivi des enfants atteints d’un Syndrome Drépanocytaire Majeur lors des visites médicales jusqu’à l’âge de 3 ans. Un protocole de traitement préventif avec la pénicilline orale a été mis en place accompagné d’une prise quotidienne d’acide folique, la vaccination, et l’hydratation. De juin 2015 à octobre 2017, 19 195 nouveau-nés ont été dépistés, et cela a permis de faire une estimation de 6 189 nouveau-nés atteints SDM par an sur le territoire national.

## Sur le conseil gÉnÉtique

Le conseil génétique vise à informer tout porteur d’un gène S, C ou ß-thalassémique de son état et du risque génétique auquel il est exposé de donner naissance à un enfant atteint d’un syndrome drépanocytaire majeur et des moyens mis à sa disposition pour faire face à ce risque. L’information sur le risque génétique n’affecte pas la personne concernée, mais sa descendance et donc que la règle du « bénéfice individuel direct » est dépassé. En effet l’étude de l’hémoglobine – test de dépistage et de diagnostic – n’est pas aujourd’hui considérée comme un « examen des caractéristiques génétiques d’un individu (test génétique) » au sens des lois de Bioéthique (France-Code de Santé Publique 2013), et il est donc nécessaire de distinguer les tests de dépistage et les tests diagnostiques. Devant le couple en consultation, il faut tenir compte des origines diverses des personnes concernées, de la barrière de la langue, du contexte familial, culturel, religieux, de la signification différente des mots selon les interlocuteurs, de l’importance de la maternité/paternité pour certains couples… Ainsi la prévention de la drépanocytose est fondée sur l’information et le dépistage des porteurs et soulève d’importantes questions d’éthique et devient un problème médico-socio-politique de Santé Publique majeur.

## Le test de diagnostic rapide

Le CEFA a présenté une étude réalisée à Kinshasa sur les performances diagnostiques du test rapide, TDR Sickle SCAN™ en comparaison avec l’Isoélectrofocalisation (IEF) dans le dépistage néonatal. Cette étude qui est la première du genre en République Démocratique du Congo, a permis de démontrer que le TDR Sickle ScanTM a une valeur intrinsèque importante et une sensibilité de 98 à 100 %, une spécificité de 100 %, et une bonne concordance entre observateurs. Cet outil simplifie considérablement le diagnostic de la drépanocytose, et peut améliorer la stratégie de diagnostic dans les conditions où la plupart des structures de prise en charge n’ont pas de chaîne de froid. Les avantages techniques et économiques de l’utilisation de la spectrométrie de masse MALDI-MS pour le dépistage de la drépanocytose à Madagascar ont été démontrés et, il est proposé d’utiliser l’approche NeoSickle - spectrométrie de masse MALDI à Madagascar; ce qui permettre de dépister aussi les prématurés dont l’âge de gestation est compris entre 24 et 26 semaines d’aménorrhée.

## Suivi et prÉvention des complications

Dans ces exposés, il a été surtout question des succès et défis dans le dépistage et suivi des drépanocytaires en Afrique subsaharienne en passant en revue les différents projets pilotes du DNN réalisés en Afrique. L’expérience béninoise a montré son succès partant du DNN, devenu quasi universel grâce à l’implication des Autorités et à un réseau de collaboration entre personnel soignant, jusqu’au suivi des enfants drépanocytaires dépistés.

## Expression clinique de la drÉpanocytose

Un travail portant sur la prévalence du déficit de la G6PD dans une série des 125 drépanocytaires suivis au CH Monkole a montré que 20 % de drépanocytaires avaient un déficit en G-6-PD mais que cette association drépanocytose et G-6-PD, n’avait pas d’influence significative sur les paramètres hématologiques. A Madagascar, un travail sur le suivi clinique et biologique des drépanocytaires sous hydroxyurée au service d’hématologie au CHU IRA d’Antananarivo, a montré la place privilégiée de l’hydroxyurée dans la prise en charge des formes sévères de la drépanocytose et la nécessité d’un suivi régulier, afin de prévenir les effets secondaires qui sont réversibles à l’arrêt du traitement. Une large étude réalisée en Afrique appelé « Étude CADRE » qui consiste à établir le lien entre l’hyper hémolyse et les complications vasculaires observées dans la drépanocytose a montré qu’aucune association n’existait entre l’hyper hémolyse chronique et les complications vasculaires attendues, Ainsi, il résulte qu’en Afrique subsaharienne, le grand marqueur du dommage vasculaire des organes semble être essentiellement le taux d’Hb.

## HydroxyurÉe et perspective

Les résultats du projet REACH (*Realizing Effectivness Across Continents with Hydroxyurea*), une étude sur l’hydroxyurée centrée sur 4 pays africains en Afrique subsaharienne [2] a démontré que l’hydroxyurée améliore sensiblement la clinique ainsi que les paramètres biologiques de la drépanocytose et réduit le risque de malaria et de complications infectieuses.

## Indications et complications liÉes À la transfusion

L’expérience de la Martinique sur la transfusion sanguine et les échanges transfusionnels montre que les indications d’une transfusion sanguine ponctuelle ainsi que des échanges transfusionnels automatisés permettent d’éviter les complications de la surcharge en fer, l’hyperviscosité et l’hypovolémie.

## L’espoir de guÉrir de la drÉpanocytose en afrique

L’expérience de la greffe de la moelle chez les patients drépanocytaires suivis à l’université d du Sultan Qaboos en Oman, a permis de guérir les patients qui avaient une expression clinique sévère ou très sévère de la drépanocytose.

## AnesthÉsie, chirurgie et drÉpanocytose

La prise en charge des complications aiguës en réanimation, en l’occurrence le syndrome thoracique aigu (STA) a permis à l’auditoire de revoir les complexes bases physiopathologiques, les pièges du diagnostic ainsi que les principes de la prise en charge.

## DrÉpanocytose et sociÉtÉ

Il est clairement démontré que les associations réunissant les malades et leurs familles contribuaient énormément dans la lutte contre cette maladie en Afrique. L’introduction, par exemple, des expressions liées à la drépanocytose dans l’Académie malgache a permis au public de mieux comprendre la maladie.

## Table ronde sur la stratÉgie de l’oms

Il est important que l’OMS s’implique davantage dans la lutte en Afrique, en appuyant les programmes nationaux qui ont été crées par les États à la suite de la reconnaissance de cette maladie comme étant une priorité de santé publique. Un état de lieux de la lutte dans les pays africains est nécessaire afin de permettre à l’OMS d’aider à mener des actions qui visent à atteindre les objectifs fixés lors des assises de MALABO (OMS, 2010).

## Nouvelles voies de recherche

L’équipe malgache de l’Institut Malgache de Recherche Appliquée (IMRA) a fait part d’une étude pharmacologique préliminaire sur des plantes à usage traditionnel dans le suivi des drépanocytaires: deux plantes ont pu montrer quelques effets bénéfiques sans avoir une toxicité pour les patients. Il faut signaler que l’équipe malgache respecte les recommandations de l’OMS dans les projets de recherche des phytomédicaments.

## Conclusion

En conclusion après les précédents symposiums du REDAC qui ont porté, entre autre, sur l’accès au traitement, les nouvelles technologies et innovations, ou encore drépanocytose et transfusion sanguine, le choix fait à Madagascar de se pencher sur les réalités pour une stratégie de lutte contre la drépanocytose dans la région africaine de l’Organisation Mondiale de la Santé, a montré le bien fondé et l’esprit du symposium inaugural organisé à Kinshasa en 2010.

## Conflits D'intérêts

Les auteurs ne déclarent aucun conflit d’intérêts.

**Fig. 2:**
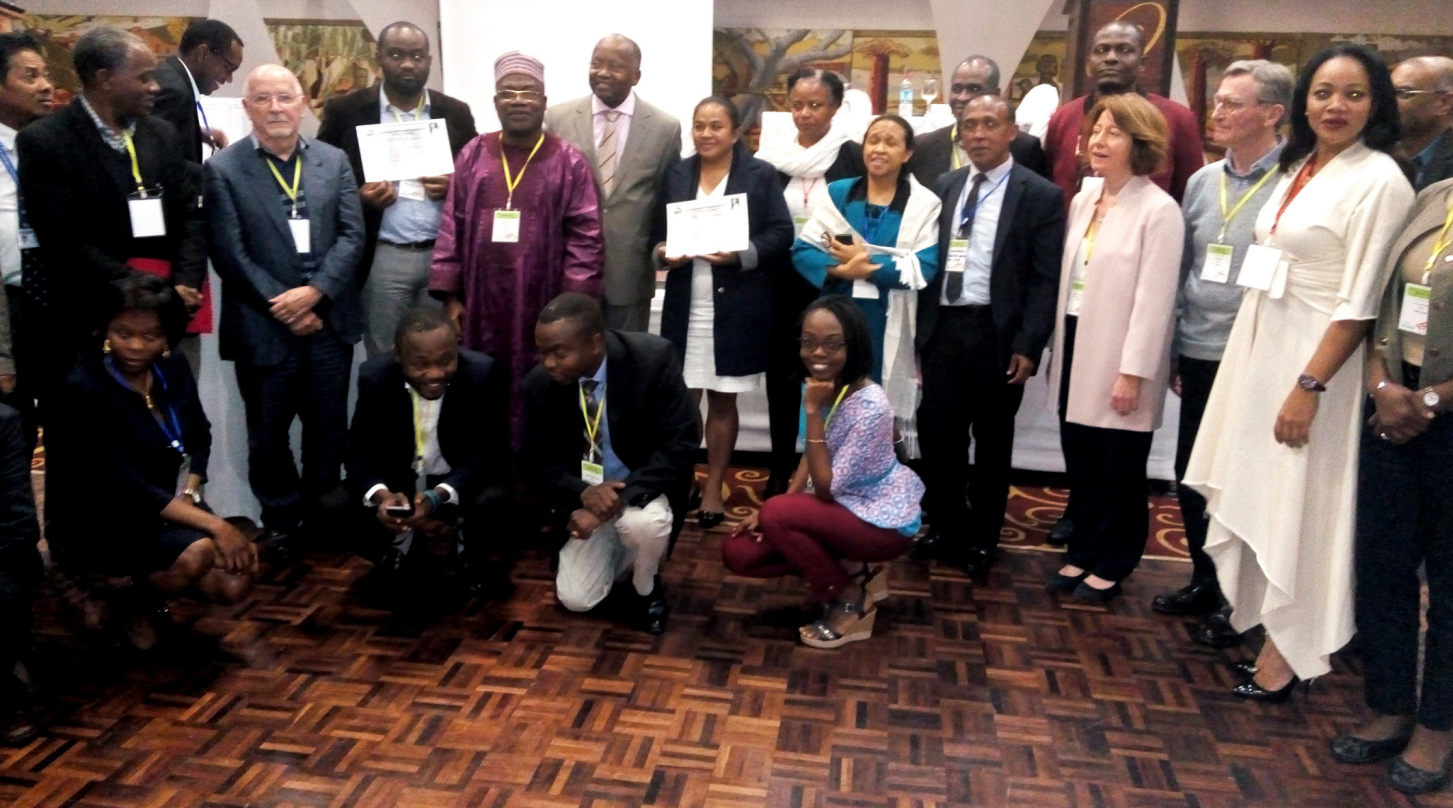
Vue sur Antanarivo depuis la salle de conférence du 7^e^ symposium du REDAC a Madagascar View of Antanarivo from the conference room of the 7^th^ symposium of the REDAC in Madagascar
